# Supraclavicular Skin Temperature as a Measure of ^18^F-FDG Uptake by BAT in Human Subjects

**DOI:** 10.1371/journal.pone.0098822

**Published:** 2014-06-12

**Authors:** Mariëtte R. Boon, Leontine E. H. Bakker, Rianne A. D. van der Linden, Lenka Pereira Arias-Bouda, Frits Smit, Hein J. Verberne, Wouter D. van Marken Lichtenbelt, Ingrid M. Jazet, Patrick C. N. Rensen

**Affiliations:** 1 Department of Endocrinology and Metabolic Diseases, Leiden University Medical Center, Leiden, The Netherlands; 2 Einthoven Laboratory for Experimental Vascular Medicine, Leiden University Medical Center, Leiden, The Netherlands; 3 Department of Nuclear Medicine, Leiden University Medical Center, Leiden, The Netherlands; 4 Department of Nuclear Medicine, Rijnland Hospital, Leiderdorp, The Netherlands; 5 Department of Nuclear Medicine, Academic Medical Center, Amsterdam, The Netherlands; 6 Department of Human Biology, NUTRIM School for Nutrition, Toxicology and Metabolism, Maastricht University Medical Center, Maastricht, The Netherlands; St. Joseph's Hospital and Medical Center, United States of America

## Abstract

**Background:**

Brown adipose tissue (BAT) has emerged as a novel player in energy homeostasis in humans and is considered a potential new target for combating obesity and related diseases. The current ‘gold standard’ for quantification of BAT volume and activity is cold-induced ^18^F-FDG uptake in BAT. However, use of this technique is limited by cost and radiation exposure. Given the fact that BAT is a thermogenic tissue, mainly located in the supraclavicular region, the aim of the current study was to investigate whether cold-induced supraclavicular skin temperature and core body temperature may be alternative markers of BAT activation in humans.

**Subjects/Methods:**

BAT volume and activity were measured in 24 healthy lean adolescent males (mean age 24.1±0.8 years), using cold-induced ^18^F-FDG uptake with PET-CT. Core body temperature was measured continuously in the small intestine with use of an ingestible telemetric capsule and skin temperature was measured by eighteen wireless iButtons attached to the skin following ISO-defined locations.

**Results:**

Proximal and distal (hand/feet) skin temperatures markedly decreased upon cold exposure, while supraclavicular skin temperature significantly increased (35.2±0.1 *vs*. 35.5±0.1°C, p = 0.001). Furthermore, cold-induced supraclavicular skin temperature positively correlated with both total (R^2^ = 0.28, P = 0.010) and clavicular BAT volume (R^2^ = 0.20, P = 0.030) and clavicular SUV_max_ (R^2^ = 0.27, P = 0.010), while core body temperature did not.

**Conclusions:**

Supraclavicular skin temperature as measured by iButtons may have predictive value for BAT detection in adult humans. This is highly desirable considering the increasing interest in pharmacological interventions to stimulate BAT in human subjects.

**Trial Registration:**

NTR 2473

## Introduction

Brown adipose tissue (BAT) is a highly metabolically active tissue involved in facultative thermogenesis in mice (reviewed in [1]) and humans [2]–[5]. Cold-induced ^18^F-fluorodeoxyglucose (FDG) positron emission tomography-computed tomography (PET-CT) studies in humans have shown that BAT is mainly present in the neck area and along the great vessels [6]. At these strategic locations, the produced heat can immediately be dispersed throughout the body. In addition, brown-like adipocytes, so-called ‘beige’ adipocytes, are present within white adipose tissue and these may contribute to total energy expenditure and thermogenesis as well, albeit to a lesser extent as the ‘classical’ brown adipocytes [7]. In order to execute their function, brown adipocytes contain a wealth of mitochondria that express uncoupling protein 1 (UCP-1), which uncouples respiration from adenosine triphosphate (ATP) synthesis, leading to heat production [1]. As a substrate, brown adipocytes oxidize triglyceride-derived fatty acids and glucose. In line with this, in mice, BAT is importantly involved in plasma triglyceride clearance [8] and contributes to glucose homeostasis [9]. Furthermore, in humans, BAT activation by means of cold induction results in elevation of plasma free fatty acids (FFA) levels and a steep increase in fat oxidation [10].

It has been estimated that fully activated BAT in humans can contribute to up to 15–20% of total energy expenditure [11]. The fact that obese individuals have lower BAT volume and activity supports the metabolic importance of BAT [6], [12], [13]. Of note, we recently showed that the South Asian population, which is highly susceptible of developing a disadvantageous metabolic phenotype along with type 2 diabetes, has markedly lower BAT volume along with lower resting energy expenditure and non-shivering thermogenesis, further supporting a role of BAT in whole-body metabolism [14]. Therefore, stimulation of BAT is currently considered a potential preventive and therapeutic target in the combat against obesity and related diseases, such as dyslipidemia and type 2 diabetes. In fact, the modest decrease in body weight as evoked by the anti-diabetic drug metformin may be due to activation of BAT [15].

The current ‘gold standard’ for determination of BAT volume and BAT activity in human subjects is cold-induced ^18^F-FDG uptake as assessed with PET-CT, for which subjects are cooled for approximately 2 hours at a temperature just above their shivering temperature [4], [12], [13], [16], followed by infusion of ^18^F-FDG and performance of a PET-CT scan. ^18^F-FDG is an analogue of glucose that is taken up by glucose transporters. Once taken up, ^18^F-FDG is phosphorylated but is no substrate for further metabolism and becomes trapped in metabolically active, glucose-using tissues, amongst which BAT, allowing the assessment of glucose uptake by tissues by PET-CT. ^18^F-FDG uptake by activated BAT regions on the PET-CT scan can be quantified, resulting in measures for BAT volume as well as for maximal ^18^F-FDG uptake (maximal standardized uptake value, ‘SUV_max_’) and mean ^18^F-FDG uptake (mean standardized uptake value, ‘SUV_mean_’), which may be considered measures for the level of BAT activation.

Interestingly, in mice, in which most BAT is present in the interscapular region, ‘interscapular temperature’ is commonly used as a measure for BAT activity [17], as well as core body temperature [18]. For instance, activation of BAT by the cannabinoid 1 receptor inverse agonist rimonabant resulted in a transient but steep increase in interscapular temperature as recorded by implantable temperature probes [17]. Since in humans a large part of BAT is located in the supraclavicular region, recording cold-induced skin temperature in this specific area may be an attractive alternative for measuring BAT activity instead of the ^18^F-FDG scans which are relatively expensive and pose a radiation burden on study subjects. Therefore, the aim of the current study was to investigate whether cold-induced supraclavicular skin temperature as measured with wireless temperature sensors and core body temperature as measured in the small intestine with an ingestible telemetric temperature sensor correlate with ^18^F-FDG uptake by BAT as measured with PET-CT imaging in lean, healthy males.

## Methods

### Subjects

24 Dutch healthy, lean (BMI <25 kg/m^2^) males of white Caucasian (n = 12) and South Asian (n = 12) origin between 18 and 28 years of age were enrolled via local advertisements as described previously [14]. Subjects underwent a medical screening including their medical history, a physical examination, blood chemistry tests and an oral glucose tolerance test to exclude individuals with type 2 diabetes according to the American Diabetes Association 2010 criteria. Other exclusion criteria were rigorous exercise, smoking and recent body weight change. The present study was approved by the Medical Ethical Committee of the Leiden University Medical Center and performed in accordance with the principles of the revised Declaration of Helsinki. All volunteers gave written informed consent before participation.

### Study design

The study was conducted in The Rijnland Hospital, Leiderdorp (The Netherlands). Subjects were studied in the morning after a 10-hour overnight fast and subjects were not allowed to exercise 24 hours prior to the study. Subjects wore standardized clothing, consisting of a T-shirt and boxer short. Upon arrival, subjects ingested a telemetric capsule to measure core body temperature, and eighteen wireless iButtons were attached to the skin to measure skin temperature (see below). A cannula was inserted in the left antecubital vein for ^18^F-FDG injection.

#### Cooling protocol

To activate BAT an individualized cooling protocol was applied, using two water perfused cooling mattresses (Blanketrol III, Cincinatti Sub-Zero (CSZ) Products, Inc), as described previously [14]. During the procedure subjects stayed in a clinical examination room (temperature approx. 24°C) in a semi-supine position. Importantly, the cooling mattresses covered the anterior and posterior sides of the body of the subject, from the caudal part of the chin and upper side of the neck, respectively, until the ankles or lower legs, depending on the height of the subject. Thus, the clavicular region of the subject was fully covered in each subject. The protocol started with a baseline period of one hour in thermoneutral condition (water temperature cooling mattresses 32°C), after which subjects were exposed to mild cold. Since the onset temperature of shivering shows a high interindividual variation (e.g. due to differences in body composition), an individualized cooling protocol was used to ensure maximal non-shivering thermogenesis (NST), and thus a maximum level of BAT activity for each subject. Cooling started at a mattress temperature of 32°C and water temperature was gradually decreased until shivering occurred. In short, we first decreased the temperature with steps of 5°C every 5 minutes. After we reached a water temperature of 17°C, we decreased the temperature with 2°C every 10 minutes. When a water temperature of 11°C was reached (but not all subjects reached this temperature), we decreased temperature with 1°C every 10 minutes. This was continued until shivering occurred. Shivering was detected visually and by asking the subject if he experienced shivering. When the shivering temperature had been reached, the subject was warmed for 3 minutes with a bathrobe so that shivering stopped and then the subject was cooled with a water temperature 3°C higher than the temperature at which shivering occurred. From that moment, the cooling period of two hours was started (t_cold_ = 0 min). In case of shivering, temperature was raised by steps of 1°C until shivering just stopped. In this manner NST was maximized for each individual without shivering. Also, just before administration of the FDG, we raised the temperature by 1°C to prevent occurrence of shivering during the FDG uptake period. During the FDG uptake period, mean inlet water temperature was 19.3°C. At the end of the first hour (t_cold_ = 60 min) of cooling ^18^F-FDG was injected intravenously (2 MBq/kg). Both in thermoneutral and cold-induced condition (t_cold_ = 110 min) indirect calorimetry was performed with a ventilated hood (Oxycon Pro, CareFusion, Germany) (t_cold_ = 80–110 min). After the second hour (t_cold_ = 120 min) of cooling ^18^F-FDG-PET-CT imaging was performed to quantify BAT.

#### ^18^F-FDG-PET-CT-scan

Imaging was performed on a PET-CT-scanner (Gemini TF PET-CT, Philips, The Netherlands) as described previously [14]. Blind to subject characteristics, both a nuclear medicine physician and two researchers analyzed the PET-CT images using dedicated software (Hermes Hybrid Viewer, Hermes Medical Solutions AB, Sweden). BAT activity and detectable BAT volume were quantified in the region of interest (as assessed by CT) by autocontouring the BAT areas with a set threshold (SUV of 2.0 g/mL).

#### Temperature registration

**Figure 1:**
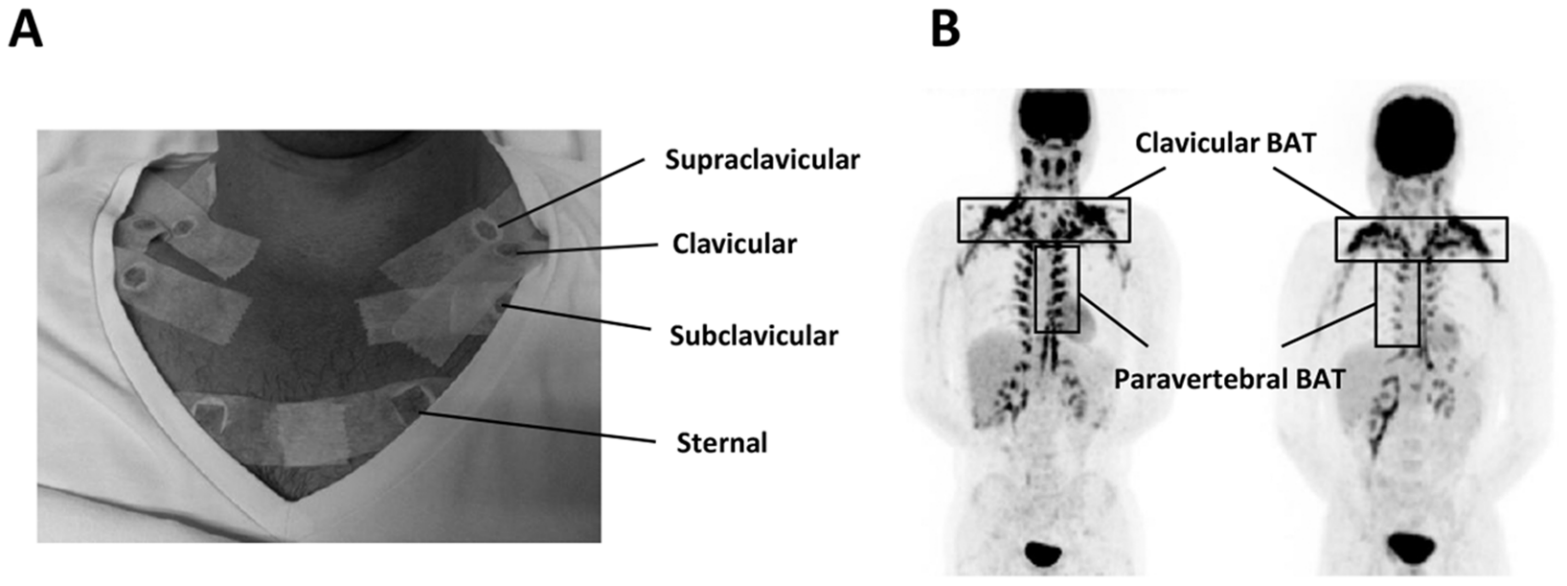
Location of iButtons and clavicular and paravertebral brown adipose tissue. In the clavicular and sternal region, iButtons were symmetrically attached at the supraclavicular, clavicular, subclavicular and sternal region (A). Clavicular and paravertebral brown adipose tissue regions in young, healthy male subjects as assessed by PET-CT scan with ^18^F-FDG (B).

Core body temperature was measured continuously in the small intestine with the use of an ingestible telemetric capsule (Jonah, BMedical, Australia) that recorded core body temperature at 1-minute intervals. Skin temperature was measured at 1-minute intervals by wireless iButtons (iButton, Maxim, USA) [19]. An iButton contains a semiconductor temperature sensor, a computer chip with a real time clock and memory, and a battery. In total eighteen iButtons were attached to the skin with adhesive tape at the following ISO-defined locations: forehead, supraclavicular (left and right), clavicular (left and right), subclavicular (left and right), sternal (left and right) (see Figure 1A), supra umbilicular, anterior thigh (left and right), lateral thigh (left and right), flat of the hand (left and right) and bow of the foot (left and right).

### Calculations

#### Total detectable BAT volume

In every slice, BAT size (measured in square centimetres) was quantified in the anatomical regions of interest (ROIs) using the auto contouring and region growing tool of the Hybrid Viewer. Detectable BAT volume (measured in millilitres) was calculated by summing up the ROIs from the individual slices, establishing a volume of interest (VOI). Since the localization of BAT differs between individuals (e.g. some have more BAT in the clavicular region, some more in the paravertebral region), both total detectable BAT volume and BAT volume in the clavicular region were quantified (Figure 1B).

#### BAT activity

Within every region of interest, the Hybrid Viewer provided two measures of ^18^F-FDG uptake, the maximal and mean standardized uptake value (SUV_max_ and SUV_mean_, respectively). The standardized uptake value (SUV) is defined as the ratio of activity [kBq per mL] within the region of interest (ROI) and the injected activity [kBq] per bodyweight [g] and is expressed in g/mL. For SUV_max,_ the highest value in the VOI was taken. For SUV_mean_ the mean value within the VOI was determined.

#### Skin temperature measurements

Distal skin temperature was calculated as the average temperature of hands and feet and proximal skin temperature as the weighted average temperature of claviculae, anterior thigh and umbilicus (Tprox = 0·383*Tavg_thighs +0·293*Tavg_clav +0·324*Tavg_umbilicus) according to the equation of Van Marken Lichtenbelt et al [19], based on the formulas by Kräuchi et al [20] and Hardy et al [21]. Mean skin temperature was calculated as the average of distal and proximal skin temperature. Core mean skin temperature gradient was calculated as the difference between core and mean skin temperature, and core distal skin temperature gradient as the difference between core and distal skin temperature. For thermoneutral skin temperature, the mean temperature of the second half of the thermoneutral period was calculated for each iButton. Cold-induced skin temperature was calculated as the mean skin temperature during the twenty minutes following ^18^F-FDG administration, i.e., at the beginning of the second hour of cooling.

### Statistical analysis

Data are presented as mean ± SEM. Paired t-tests were used to assess mean differences before and after cold exposure. To identify correlations between variables, linear regression analyses were performed. Significance level was set at P<0.05. Statistical analyses were performed using SPSS for Windows version 20.0 (IBM, USA).

## Results

### Clinical characteristics

Clinical characteristics are shown in Table 1. Part of these data have recently been published for white Caucasians and South Asians separately [14]. However, analysis of covariance showed that there was no interaction between ethnicity and BAT volume with supraclavicular temperature as dependent factor (p = 0.36) and that there was no difference in the intercept between South Asian and white Caucasian subjects (p = 0.33). We, therefore, pooled the data for both ethnicities. One subject developed hyperventilation following ^18^F-FDG administration, and was therefore excluded from all cold-induced and BAT measurements. Mean age was 24.1±0.6 years and mean BMI was 21.7±0.4 kg/cm^2^. Systolic and diastolic blood pressure were significantly increased in response to cold (119±2 *vs*. 134±4 mmHg, p = 0.00002; 66±1 *vs*. 82±2 mmHg, p = 0.000000), while heart rate was not affected.

**Table 1:** Clinical characteristics in young, healthy, male subjects.

	Healthy male subjects (n = 24)	
	Thermoneutral	Cold-induced
age (years)	24.1±0.6	
body mass index (kg/m^2^)	21.7±0.4	
systolic blood pressure (mmHg)	119±2	134±4**
diastolic blood pressure (mmHg)	66±1	82±2**
heart rate (bpm)	60±2	57±2
SUV_max_ (g/mL)		15±1.0
SUV_mean_ (g/mL)		4.1±0.1
Total BAT volume (mL)		235±29
Clavicular SUV_max_ (g/mL)		14±1
Clavicular SUV_mean_ (g/mL)		4.6±1
Clavicular BAT volume (mL)		84±1
Resting energy expenditure (kcal/day)	1493±52	1732±91**

Data are presented as mean ± SEM. **p<0.005 *vs*. thermoneutral condition.

In all subjects active BAT was detected, as evidenced by ^18^F-FDG uptake in the classical BAT regions, though one subject exhibited only 1.5 mL of detectable BAT. Mean total BAT volume was 235±29 mL. Mean SUV_max_ was 15±1 g/mL, and mean SUV_mean_ was 4.1±0.1 g/mL for total BAT volume. In the clavicular region, mean BAT volume was 84±11 mL. Mean SUV_max_ in this region was 14±1 g/mL, while mean SUV_mean_ was 4.6±0.1 g/mL. Cold stimulation resulted in an increase in energy expenditure, i.e. nonshivering thermogenesis, by 16% (16±4%, p = 0.001).

### Thermoregulation

Thermoregulation is shown in table 2. Mean inlet water temperature of the cooling matresses at which shivering started was 9.9±0.4°C. Mean core body temperature was not affected by cold exposure. Mean total, proximal and distal skin temperature markedly decreased on cold stimulation. Consequently, core distal and core mean skin temperature gradients were significantly higher during cooling, indicating an insulative response. With respect to the clavicular region, supraclavicular skin temperature was significantly increased upon cold exposure (35.2±0.1 *vs*. 35.5±0.1°C, p = 0.001). Clavicular skin temperature was not affected and subclavicular skin temperature was significantly reduced.

**Table 2:** Thermoregulation in thermoneutral and cold-induced condition in young, healthy, male subjects.

	Healthy male subjects (n = 24)	
	Thermoneutral	Cold-induced
shiver temp		9.9±0.4
core body temp	36.7±0.1	36.7±0.1
mean skin temp	33.3±0.1	28.7±0.2**
mean proximal skin temp	34.4±0.1	30.9±0.2**
supraclavicular skin temp	35.2±0.1	35.5±0.1**
clavicular skin temp	34.4±0.1	34.2±0.2
subclavicular skin temp	34.4±0.1	33.6±0.2**
mean distal skin temp	32.3±0.2	26.5±0.3**
forehead skin temp	33.7±0.1	33.2±0.1**
distal proximal skin temp gradient	−2.2±0.2	−4.4±0.3**
core mean skin temp gradient	3.3±0.1	8.0±0.2**
core distal skin temp gradient	4.4±0.3	10.1 ±0.3**
core proximal skin temp gradient	2.3±0.1	5.9±0.2**

Data are presented as mean ± SEM. Units are in degrees Celsius. **p<0.005 *vs*. thermoneutral condition. Temp, temperature.

### Correlations thermoregulation and ^18^F-FDG uptake by BAT

**Figure 2:**
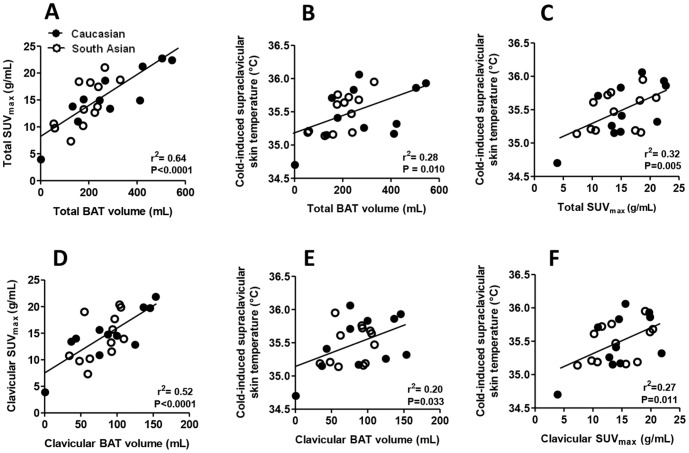
Correlations between cold-induced supraclavicular skin temperature and ^18^F-FDG uptake by brown adipose tissue in young, healthy male subjects. Total SUV_max_ in relation to total BAT volume (A). Cold-induced supraclavicular skin temperature in relation to total BAT volume (B) and total SUV_max_ (C). Clavicular SUV_max_ in relation to clavicular BAT volume (D). Cold-induced supraclavicular skin temperature in relation to clavicular BAT volume (E) and clavicular SUV_max_ (F). Correlations were determined by linear regression analysis. Open circles represent South Asian subjects, black circles white Caucasian subjects. BAT, brown adipose tissue. SUV, standard uptake value.

**Figure 3:**
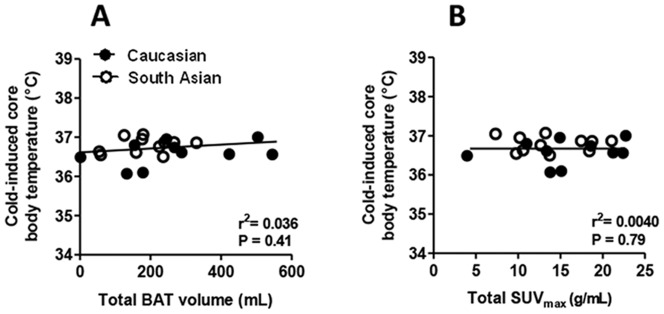
Correlations between cold-induced core body temperature and ^18^F-FDG uptake by brown adipose tissue in young, healthy male subjects. Cold-induced core body temperature in relation to total BAT volume (A) and total SUV_max_ (B). Correlations were determined by linear regression analysis. Open circles represent South Asian subjects, black circles white Caucasian subjects. BAT, brown adipose tissue. SUV, standard uptake value.

Linear regression analysis showed a clear positive correlation between SUV_max_ and total BAT volume (R^2^ = 0.64, P<0.0001) (Figure 2A; this figure is modified from a previous publication [14]). Furthermore, a positive correlation was found between cold-induced supraclavicular skin temperature and total BAT volume (R^2^ = 0.28, P = 0.010) (Figure 2B) and SUV_max_ (R^2^ = 0.32, P = 0.005) (Figure 2C). The positive correlation remained after removal of the subject with the lowest detectable BAT volume (R^2^ = 0.18, P = 0.049 and R^2^ = 0.18, P = 0.047 for total BAT volume and SUV_max_, respectively). With respect to BAT that was located in the clavicular region only, again a clear positive correlation was found between SUV_max_ and BAT volume (R^2^ = 0.52, P<0.0001) (Figure 2D). Furthermore, cold-induced supraclavicular skin temperature correlated positively with clavicular BAT volume (R^2^ = 0.20, P = 0.030) (Figure 2E) and clavicular SUV_max_ (R^2^ = 0.27, P = 0.010) (Figure 2F). No correlations were found between cold-induced clavicular and subclavicular skin temperature and total and clavicular BAT volume (data not shown). Furthermore, both total BAT volume and SUV_max_ did not correlate with cold-induced core body temperature (R^2^ = 0.04, P = 0.410, Figure 3A; R^2^ = 0.004, P = 0.790, Figure 3B; respectively). Finally, no correlations were found between delta (i.e. the change in temperature upon cooling) supraclavicular, clavicular and subclavicular skin temperature and total and clavicular BAT volume, respectively (data not shown).

## Discussion

BAT has emerged as a novel player in energy homeostasis in humans and is currently considered a potential new target for obesity and related diseases. The current ‘gold standard’ for quantification of BAT volume and activity in humans is the cold-induced ^18^F-FDG uptake measured with PET-CT, which is, however, limited by cost and radiation exposure. Therefore, a less expensive and less burdensome alternative to determine BAT in human research is highly desirable. In the present study, we demonstrated that, while mean proximal and distal skin temperatures were markedly decreased upon cold exposure as expected, supraclavicular skin temperature was significantly increased. Furthermore, cold-induced supraclavicular skin temperature was positively correlated with both total and clavicular BAT volume and SUV_max_, suggesting that cold-induced supraclavicular skin temperature may have predictive value for BAT detection in humans. BAT is a thermogenic tissue with special importance in neonates who are sensitive to develop hypothermia due to their large body surface area [1]. In this study, we detected BAT in 100% of the subjects with a corresponding thermogenic response, as indicated by a mean increase in energy expenditure of 16%. The strategic location of BAT, mainly in the interscapular region in neonates and in the neck area and along the great vessels in adults, results in efficient spreading of the produced heat throughout the body. Hence, an increase in temperature of the skin that overlies BAT, i.e. the supraclavicular region, upon BAT activation may be expected. Heat production by BAT is the direct result of uncoupling of ATP synthesis by the uncoupling protein UCP-1. The main stimulus for activation of this intracellular cascade is sympathetic activation with subsequent release of catecholamines, i.e. following cold induction [1]. Next to heat production, sympathetic activation may also result in alterations in BAT blood flow [2], aimed at spreading the heat throughout the body to maintain core body temperature and away from BAT. In addition, sympathetic activation induces peripheral cutaneous vasoconstriction [22], also facilitating maintenance of core body temperature. In the present study, blood pressure and core distal and core mean skin temperature gradients were significantly increased during cooling, suggesting cold-induced activation of the sympathetic nervous system.

We found that supraclavicular skin temperature significantly increased upon cold exposure. This is in line with a recent study in healthy human volunteers in which short-term (5 minutes) cooling of the hand resulted in a highly localized increase in supraclavicular skin temperature as measured by infrared thermal imaging [23]. However, in that study, changes in skin surface temperature had not been directly correlated with direct measurements of BAT activity. Rather, PET-CT images from a comparable group of age- and sex matched subjects were used. In the current study, we combined skin temperature measurements with ^18^F-FDG PET-CT scans in the same subjects and found that cold-induced supraclavicular skin temperature significantly correlated with clavicular BAT volume and clavicular SUV_max_, though the R^2^ was modest (up to 0.27 for clavicular SUV_max_). Moreover, a comparable correlation was found with total BAT volume and SUV_max_ in the total BAT region. Thus, supraclavicular skin temperature may not only be indicative for activated BAT present in the clavicular region, but also for the total amount of BAT. Interestingly, Yoneshiro et al [5] previously showed that supraclavicular skin temperature as measured by means of small disc-type temperature data loggers was only decreased in subjects without detectable BAT during cold while in subjects with detectable BAT it remained equal. This difference may be attributable due to a difference in thermogenesis between BAT positive and BAT negative subjects. Our results are furthermore in accordance with a previous mouse study in which changes in BAT temperature as determined by thermal imaging highly correlated with increases in ^18^F-FDG uptake within BAT [24]. Of note, we used iButtons to measure skin temperature instead of thermal imaging. iButtons have been shown to be a reliable, safe, cheap and extensively researched technique in both cold and warm conditions [19].

The rise in supraclavicular skin temperature may not solely be due to thermogenesis by activated BAT, but also due to short-term changes in BAT blood flow, as has been shown in rats [25] as well as in humans [2]. In line with this, cold acclimation in mice leads to increased mitochondrial density and increased sympathetic nerve fiber density together with increased angiogenesis [26]. The physiological role of this increased blood flow is likely to spread the heat throughout the body to maintain core body temperature. Thus, the increased supraclavicular skin temperature is likely the consequence of a combination of increased BAT thermogenesis as well as increased BAT blood flow. However, since the underlying mechanism (i.e. increased sympathetic activation towards BAT) is the same, both provide a good measure of BAT activation and may thus be measured at the skin surface.

In our study, clavicular and subclavicular skin temperature did not correlate with BAT volume. This is likely explained by the fact that most of the clavicular BAT pool is located in the supraclavicular region ([23], and see Figure 1). Furthermore, core body temperature did not correlate with total BAT volume nor with total SUV_max_. In mice, core body temperature is commonly used as a measure for BAT activity [17], and core body temperature may transiently rise by as much as 1.5°C following BAT activation. However, as is evident from the current study as well as from a previous study [12], in human subjects core body temperature does not rise following BAT activation due to cold exposure but rather stays equal, likely as a consequence of BAT activation preventing a drop in core body temperature. The lack of a rise in core body temperature could be due to the fact that the relative amount of BAT in humans is lower as compared to mice. Accordingly, in mice housed at 5°C nutrient oxidation in BAT can account for over 60% of the total energy expenditure [27], [28], as compared to 15–20% in humans [11], resulting in tremendous heat production and a subsequent rise in core body temperature. Thus, in humans, cold-induced core body temperature is likely no good measure of BAT volume.

A potential limitation of the current study is our cooling protocol. We used a personalized cooling protocol in which water-perfused cooling mattresses were used to cool subjects just above their shiver temperature. Though this cooling protocol results in maximal BAT activation under non-shivering conditions [11], the cooling mattresses do not cover all parts of the body, such as the head and feet. Furthermore, since the supraclavicular region is located at the upper region of the mattresses, we cannot exclude that this region may have been influenced by warmer airflows. However, temperature of the forehead, which was not cooled, significantly decreased upon the cooling protocol making influence of warmer airflows less likely. Furthermore, an extra bed sheet covered the upper cooling mattress in order to create a cold compartment. Lastly, in the study by Symonds et al [23], short-term cooling of a hand or foot also resulted in a rise in supraclavicular skin temperature, supporting the concept that a cold stimulus induces rapid BAT activation accompanied by supraclavicular heat production. Furthermore, we acknowledge that the predictive value of cold-induced supraclavicular skin temperature is limited, with a maximum of 32% for the prediction of total SUV_max._ Thus, further studies are needed to confirm our results and to fine tune the current methods. A strength of our study is that changes in supraclavicular skin temperature were correlated with measures of BAT activity as measured by ^18^F-FDG uptake in BAT. Furthermore, we measured BAT volume and SUV_max_ in both the total detectable BAT region and the clavicular region, in order to prevent confounding by localization.

In conclusion, supraclavicular skin temperature as measured by iButtons is a potential novel non-invasive tool that may have predictive value for BAT detection in adult humans. This is highly desirable, since there is increasing interest in pharmacological interventions to stimulate BAT in human subjects given its role in energy homeostasis. Therefore, supraclavicular skin temperature may be a non-invasive method to monitor BAT activity in response to treatments.
